# Parental Guilt as a Mediator Between Self-Efficacy and Adjustment Disorders in Pediatric Oncology

**DOI:** 10.1192/j.eurpsy.2026.11554

**Published:** 2026-07-31

**Authors:** S. Elbaz, M. Kestler-Peleg, M. Kagan

**Affiliations:** Social Work, Ariel University, Ariel, Israel

## Abstract

**Introduction:**

Childhood cancer is one of the most significant crises parents may face in their lifetime. Such diagnoses dramatically alter life, introducing sudden unpredictability and lack of control, with significant psychological consequences including distress, fear of their child’s death, anxiety, depression, and impaired psychological resilience. Under these challenging circumstances, parental roles undergo substantial transformation, requiring parents to expand their responsibilities beyond traditional parenting. These demands can significantly impact parental self-efficacy. Lower levels of parental self-efficacy may lead to feelings of helplessness and increased parenting stress, potentially contributing to the development of adjustment disorder. Adjustment disorder has been identified as the second most common mental health issue among parents of children with cancer, following depression. However, the mechanisms underlying the relationship between parental self-efficacy and adjustment disorder remain poorly understood, particularly regarding the role of parental guilt as a potential mediator in this association.

**Objectives:**

Guided by transactional stress theory, this study explored parental guilt as a mediator between parental self-efficacy and adjustment disorder among parents of children with cancer. The study seeks to inform the development of targeted interventions and support services designed to enhance parental self-efficacy, reduce parental guilt, and improve parents’ mental health.

**Methods:**

A sample of 218 Israeli parents of children with cancer completed these self-report questionnaires.

**Results:**

The findings revealed that parental guilt to fully mediated the association between parental self-efficacy and adjustment disorder, such that higher levels of parental self-efficacy were associated with lower levels of parental guilt, which, in turn, were associated with lower levels of parents’ adjustment disorder.

**Conclusions:**

Policymakers and healthcare professionals should address parental experiences and promote parental self-efficacy, a core component of parental identity. This can reduce parental guilt and parents’ adjustment disorder. Parents of children with cancer should be viewed as an integral part of the treatment system and offered appropriate support. Raising awareness of the unique challenges faced by these parents can help reduce the stigma associated with seeking mental health help and encourage help-seeking behaviors. Healthcare providers should also be aware of the potential for significant parental guilt among parents of children with cancer. By using empathetic language, acknowledging the limitations of parental control, and providing support services, parental guilt may be reduced, leading to improved outcomes for both parents and children.

**Disclosure of Interest:**

None Declared

